# Heart Rate Variability is Related to Disease Severity in Children and Young Adults with Pulmonary Hypertension

**DOI:** 10.3389/fped.2015.00063

**Published:** 2015-07-07

**Authors:** Heiner Latus, Dirk Bandorski, Friederike Rink, Henning Tiede, Jannos Siaplaouras, Ardeschir Ghofrani, Werner Seeger, Dietmar Schranz, Christian Apitz

**Affiliations:** ^1^Pediatric Heart Centre, University Children’s Hospital Giessen, Giessen, Germany; ^2^German Centre for Lung Research, University of Giessen and Marburg Lung Centre, Giessen, Germany; ^3^Department of Pediatric Cardiology, University Children’s Hospital Ulm, Ulm, Germany

**Keywords:** heart rate variability, pulmonary hypertension, pediatrics, pediatric cardiology, Holter electrocardiogram, arrhythmias

## Abstract

**Background:**

Pulmonary hypertension (PH) is frequently associated with an increase in sympathetic tone. This may adversely affect cardiac autonomic control. Knowledge about the clinical impact of autonomic dysfunction in patients with PH is limited. We aimed to assess whether parameters of heart rate variability (HRV) are related to disease severity in children with PH.

**Methods:**

Parameters of HRV [SDNN, standard deviation of normal-to-normal intervals and SDANN, standard deviation of mean values for normal-to-normal intervals over 5 min] were determined from Holter electrocardiograms of 17 patients with PH without active intracardial shunt (10 female, mean age 12.8 ± 8.7 years). Patients were allocated to two groups according to their disease severity: patients with moderate PH [ratio of pulmonary to systemic arterial pressure (PAP/SAP ratio) < 0.75] (*n* = 11), patients with severe PH (PAP/SAP ratio > 0.75) (*n* = 6). An additional group of five adolescents with Eisenmenger syndrome (PAP/SAP ratio 1.13 ± 0.36) was included.

**Results:**

Children with severe PH had significantly lower values of HRV [SDNN (73.8 ± 21.1 vs. 164.9 ± 38.1 ms), SDANN (62.2 ± 19.0 vs. 139.5 ± 33.3 ms)] compared to patients with moderate PH (*p* = 0.0001 for all). SDNN inversely correlated with ratio of PAP/SAP of PH patients without shunt (*r* = −0.82; *p* = 0.0002). Eisenmenger patients showed no significant difference of HRV [SDNN 157.6 ± 43.2 ms, SDANN 141.2 ± 45.3 ms] compared to patients with moderate PH without shunt (*p* > 0.05 for all).

**Conclusion:**

According to our results, children with severe PH may have alterations in HRV. Since HRV appears to be related to disease severity, it may therefore serve as an additional diagnostic marker of PH. Remarkably, although Eisenmenger patients have systemic pulmonary arterial pressures, they seem to have preserved HRV, which might reflect a more favorable autonomic adaptation.

## Introduction

Pulmonary hypertension (PH) is characterized by progressive pulmonary vascular remodeling and consecutive elevation of pulmonary vascular resistance and pressure, which may ultimately result in right ventricular failure and death. Chronic right heart failure is associated with an increase in sympathetic tone, which may adversely affect cardiac autonomic control (1, 2). Little is known about autonomic dysfunction in patients with PH. The analysis of heart rate variability (HRV) as a marker of autonomic dysfunction can be obtained from conventional Holter-ECG recordings, which are frequently performed as part of the routine care in pediatric cardiology. An abnormal HRV has been shown in adults and children with pulmonary arterial hypertension (PAH) and seems to be associated with poor outcome (1–3). An abnormal HRV has also been shown to be an independent predictor of adverse prognosis in pediatric patients with different forms of congenital heart disease (4–6).

Since PAH is a progressive disease and shows a high mortality, despite new developments in the treatment of PAH in children (7), markers of disease severity to identify patients at risk are required and may provide early detection of clinical worsening or help to guide medical therapy (8).

The aim of our study was therefore to assess whether sympathetic parameters of HRV are related to disease severity in children with PH.

## Patients and Methods

Seventeen patients with PH (10 female, mean age 12.8 ± 8.7 years) were included in this prospective study after written informed consent was obtained. Twelve of the 17 patients had idiopathic PAH, 3 patients had associated chronic lung disease, 2 had “out of proportion” PH due to left heart disease (pulmonary vein stenosis in one, and borderline left ventricle in another one). An additional group of five adolescents with Eisenmenger syndrome was studied. A cohort of 10 healthy children, adolescents, and young adults (mean age 14.8 ± 11.2 years; 6 females) served as a control group.

Holter-ECG recording (CardioMem^®^CM 3000, GETEMED, Teltow, Germany) was performed for 48 h during daily activities. None of the patients had a history of palpitations or syncope. Data were analyzed by an operator-controlled analysis software package (CardioDay^®^, GETEMED, Teltow, Germany). Parameters of HRV (SDNN, standard deviation of normal-to-normal intervals, SDANN, standard deviation of mean values for normal-to-normal intervals over 5 min, rMSSD, square root of the mean square differences of successive RR intervals, and pNN50, proportion of the number of pairs of successive normal-to-normal intervals that differ by more than 50 ms divided by total number of normal-to-normal interval) were determined from the recordings.

The clinical data obtained included WHO functional class, echocardiographic assessment of tricuspid annular plane systolic excursion (TAPSE), and right atrial area indexed for body surface area, and at cardiac catheterization, right atrial pressure (RAP), mean pulmonary arterial pressure (mPAP), ratio of mPAP to mean systemic arterial pressure (mSAP), pulmonary capillary wedge pressure (PCWP), mixed venous oxygen saturation (mixed venous sO_2_), cardiac index (CI) (using Fick method), and pulmonary vascular resistance index (PVRI). Analysis of invasive hemodynamic data has been performed retrospectively, time interval between cardiac catheterization and Holter-ECG was 18 ± 14 months. Cardiac catheterization was performed free-breathing under sedation with midazolam and propofol.

Data from all the children were analyzed and then data from those with moderate (defined as an mPAP/mSAP ratio of <0.75) and severe PH (mPAP/mSAP ratio >0.75) were analyzed separately. The study was approved by the local Ethics Committee.

### Statistical analysis

The data are presented as mean and SDs. Differences between groups were examined for significance by the use of unpaired *t*-test and non-parametric Mann–Whitney *U*-test as appropriate. Correlations were tested using linear regression analysis. Analysis was performed using GraphPad statistical software package (San Diego, CA, USA). A *p* value ≤0.05 was considered statistically significant.

## Results

According to their disease severity, patients could be allocated to two groups: (group 1) patients with moderate PH (mPAP/mSAP ratio <0.75) (*n* = 11) and (group 2) patients with severe PH (PAP/SAP ratio >0.75) (*n* = 6). There were no significant differences in age, time interval since diagnosis, height, weight, and body surface area between both groups (Table 1). About 81% of patients of group 1 were in WHO functional class I and II, whereas 67% of patients in group 2 were in WHO functional class III and IV. Idiopathic PAH was the most frequent diagnosis (73 and 67%) in both groups. All patients received PAH-specific medication, 13 patients as combination therapy. In addition, two patients (one of each group) received digoxin, and one patient in group 2 was on betablocker therapy.

**Table 1:** **Patient characteristics**.

	All patients	PAP/SAP < 0.75	PAP/SAP > 0.75	Significance *p*
Age (years)	12.8 ± 8.7	13.7 ± 7.7	11.1 ± 11.0	0.58
Time interval since diagnosis (months)	40.5 ± 31.3	43.6 ± 27.2	34.6 ± 39.8	0.588
Height (cm)	132.8 ± 36.3	143.9 ± 28.2	112.3 ± 43.0	0.086
Weight (kg)	36.3 ± 26.1	42.6 ± 26.7	24.9 ± 22.6	0.19
BSA (m^2^)	1.13 ± 0.6	1.28 ± 0.5	0.86 ± 0.6	0.14
WHO functional class (I/II/III/IV)	2/9/5/1	2/7/2/0	–/2/3/1	
Gender (female/male)	10/7	6/5	4/2	
Diagnosis IPAH *n* (of total)	12/17	8/11	4/6	
PAH-specific medication (PDE5i/ERA/inhProstanoid/CCB)	14/8/2/4	8/4/2/4	6/4/0/0	
Combination therapy (dual/triple)	13/0	9/0	4/0	
Antiarrhythmic medication (BB/Digoxin)	1/2	0/1	1/1	

*PDE-5i, phosphodiesterase-5 inhibitor; ERA, endothelin receptor antagonist; CCB, calcium-channel-blocker; BB, betablocker; BSA, body surface area; WHO, World Health Organization*.

### Echocardiographic and hemodynamic measurements

RV systolic function assessed by TAPSE was impaired (*Z*-score < −2) in three patients in group 1 (27%) and five patients in group 2 (83%), resulting in significantly worse TAPSE *Z*-score in patients with severe PH (−0.48 ± 2.7 vs. −3.59 ± 1.5; *p* = 0.02) (Table 2). Patients with severe PH also had larger right atrial dimensions assessed by right atrial area indexed for body surface area compared to patients with mild PH.

**Table 2:** **Echocardiographic and hemodynamic measurements**.

	All patients	PAP/SAP < 0.75	PAP/SAP > 0.75	Significance *p*
TAPSE (*Z*-score)	-1.64 ± 2.7	-0.48 ± 2.7	-3.59 ± 1.5	0.02
RA-area/BSA	12.4 ± 4.2	9.9 ± 1.8	14.9 ± 4.4	0.025
RAP (mmHg)	5.4 ± 2.9	5.1 ± 3.4	5.8 ± 2.2	0.69
mPAP (mmHg)	48.4 ± 17.3	39.2 ± 12.4	63.7 ± 13.2	0.002
mPAP/mSAP	0.62 ± 0.26	0.47 ± 0.15	0.91 ± 0.18	0.0002
PCWP (mmHg)	8.9 ± 3.6	7.1 ± 3.0	11.8 ± 2.6	0.006
Hb (g/dl)	12.6 ± 2.2	12.6 ± 2.6	12.7 ± 1.7	0.92
Mixed venous sO_2_ (%)	69.9 ± 7.4	72.6 ± 7.0	65.8 ± 6.5	0.08
CI (l/min/m^2^)	3.9 ± 1.2	4.1 ± 1.2	3.7 ± 1.3	0.56
PVRI (WUxm^2^)	14.0 ± 7.9	10.5 ± 5.4	21.1 ± 7.8	0.008

*TAPSE, tricuspid annular plane systolic excursion; RA, right atrium; BSA, body surface area; RAP, right atrial pressure; mPAP, mean pulmonary arterial pressure; mSAP, mean systemic arterial pressure; PCWP, pulmonary capillary wedge pressure; Hb, hemoglobin; CI, cardiac index; venous sO_2_, venous oxygen saturation; PVRI, pulmonary vascular resistance index*.

Right atrial pressure was not elevated for the whole group (5.4 ± 2.9 mmHg) and was not significantly different between both subgroups. Mean PAP for all children was 48.4 ± 17.3 mmHg, mPAP/mSAP 0.62 ± 0.26, and PVRI 14.0 ± 7.9 WUxm^2^, and all of these measures showed significant differences between both subgroups. PCWP was significantly higher in group 2 (11.8 ± 2.6 mmHg) compared to 7.1 ± 3.0 mmHg in group 1, which can be explained by the fact that both patients with “out of proportion” PH due to left heart disease were in group 2. CI and mixed venous sO_2_ was lower in group 2, however, did not reach statistical significance (Table 2).

### Holter-ECG

Minimal heart rate (HR) was significantly higher in children with severe PH (75.7 ± 12.2 bpm) compared to children with moderate PH (56.5 ± 10.3 bpm) (*p* = 0.004). Mean HR was also higher in patients of group 2 compared to group 1, but did not reach statistical significance. In contrast, maximum HR tended to be lower in patients of group 2 (Table 3). Children of group 2 had significantly lower values of HRV [SDNN (73.8 ± 21.1 vs. 164.9 ± 38.1 ms), SDANN (62.2 ± 19.0 vs. 139.5 ± 33.3 ms), rMSSD (31.0 ± 8.7 vs. 73.6 ± 22.7 ms), and pNN50 (5.8 ± 3.4 vs. 28.0 ± 8.8%)] compared to group 1 (*p* = 0.0001 for all).

**Table 3:** **Holter monitoring data**.

	All patients	PAP/SAP < 0.75	PAP/SAP > 0.75	Significance *p*	Control group	Significance p*
Mean HR (bpm)	88.4 ± 17.5	83.5 ± 15.2	97.3 ± 19.1	0.12	85.9 ± 11.3	0.69; 0.15
Min HR (bpm)	63.3 ± 14.2	56.5 ± 10.3	75.7 ± 12.2	0.004	58.8 ± 12.7	0.86; 0.01
Max HR (bpm)	148.4 ± 32.5	156.4 ± 30.7	133.7 ± 33.0	0.18	167.4 ± 19.4	0.35; 0.02
SDNN (ms)	132.8 ± 55.3	164.9 ± 38.1	73.8 ± 21.1	0.0001	181.1 ± 43.9	0.39; 0.001
SDANN (ms)	112.2 ± 47.5	139.5 ± 33.3	62.2 ± 19.0	0.0001	152.1 ± 40.2	0.45; 0.0003
rmSSD (ms)	58.6 ± 28.0	73.6 ± 22.7	31.0 ± 8.7	0.0001	75.1 ± 34.5	0.91; 0.01
pNN50 (%)	20.2 ± 13.1	28.0 ± 8.8	5.8 ± 3.4	0.0001	22.8 ± 12.5	0.30; 0.008

*PAP, pulmonary arterial pressure; SAP, systemic arterial pressure; HR, heart rate (per minute; min = minimum, max = maximum); SDNN, standard deviation of normal-to-normal intervals; SDANN, standard deviation of mean values for normal-to-normal intervals over 5 min; rMSSD, square root of the mean square differences of successive RR intervals; pNN50, proportion of the number of pairs of successive normal-to-normal intervals that differ by more than 50 ms divided by total number of normal-to-normal interval*.

*p = PAP/SAP < 0.75 vs. controls; PAP/SAP > 0.75 vs. controls.

While no differences in measures of HRV were found between patients with moderate PH and the control group, all values of HRV (SDNN, SDANN, rMSSD, and pNN50) were significantly lower in patients with severe PH compared to healthy controls (Table 3).

### Arrhythmias

Supraventricular premature beats were recorded in five patients of group 1 and two patients of group 2, whereas only two patients had more than three premature beats per hour. There was no occurrence of atrial tachycardia or atrial flutter. Ventricular premature beats were recorded in four patients of group 1 and one of group 2, whereas only one patient had more than three premature beats per hour. There was no occurrence of higher degreed ventricular arrhythmias. No relation between the occurrence of arrhythmias and disease severity could be seen.

### Correlations

**Figure 1:**
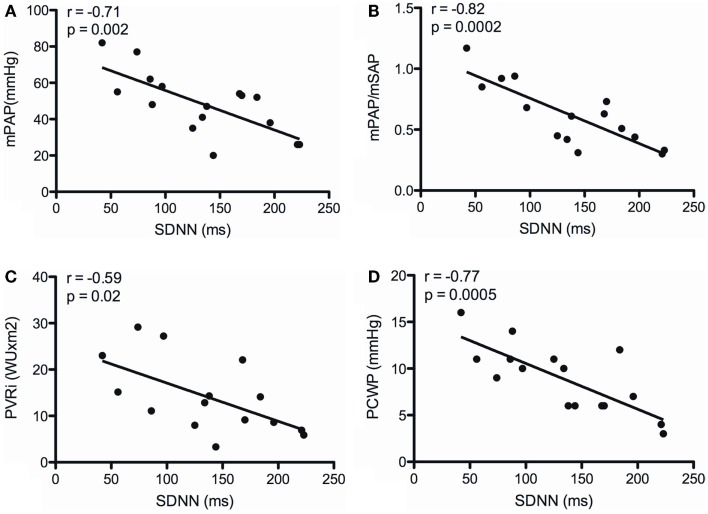
**Correlations between the SD of normal-to-normal intervals (SDNN) and mean pulmonary arterial pressure (mPAP) (A), the ratio of mean pulmonary arterial to systemic arterial pressure (mPAP/mSAP) (B), indexed pulmonary vascular resistance (PVRI) (C), and pulmonary capillary wedge pressure (PCWP) (D)**.

There was no correlation between SDNN and TAPSE, as well as indexed right atrial area. SDNN neither correlated with RAP, Hb, mixed venous sO_2_, and CI (Table 4). However, SDNN correlated inversely with mPAP (*r* = −0.71; *p* = 0.002), the ratio of PAP/SAP (*r* = −0.82; *p* = 0.0002), PVRI (*r* = −0.59; *p* = 0.02), and PCWP (*r* = −0.77; *p* = 0.0005) (Figure 1).

**Table 4:** **Correlations with SDNN**.

	Correlation *r*	Significance *p*
TAPSE (*Z*-score)	0.35	0.19
RA-area/BSA	−0.49	0.11
RAP (mmHg)	−0.37	0.19
mPAP (mmHg)	−0.71	0.002
mPAP/mSAP	−0.82	0.0002
PCWP (mmHg)	−0.77	0.0005
Hb (g/dl)	0.05	0.86
Mixed venous sO_2_ (%)	0.42	0.11
CI (l/min/m^2^)	0.05	0.88
PVRI (WUxm^2^)	−0.59	0.02

*TAPSE, tricuspid annular plane systolic excursion; RA, right atrium; BSA, body surface area; RAP, right atrial pressure; mPAP, mean pulmonary arterial pressure; mSAP, mean systemic arterial pressure; PCWP, pulmonary capillary wedge pressure; Hb, hemoglobin; CI, cardiac index; venous sO_2_, venous oxygen saturation; PVRI, pulmonary vascular resistance index*.

### Eisenmenger syndrome

Patients with Eisenmenger syndrome were significantly older (25.1 ± 5.2 years) compared to patients of group 1 and 2. All of them were in WHO functional class II and III and all of them were on PAH specific medications. Right ventricular function was preserved (TAPSE *Z*-value −0.65) and indexed right atrial area was 11.01 ± 3.38 cm^2^/m^2^. RAP was 9.0 ± 5.7 mmHg, mPAP 91.3 ± 30.6 mmHg, ratio of mPAP/mSAP was 1.13 ± 0.36 and PVRI 33.8 ± 15.9 WUxm^2^.

**Figure 2:**
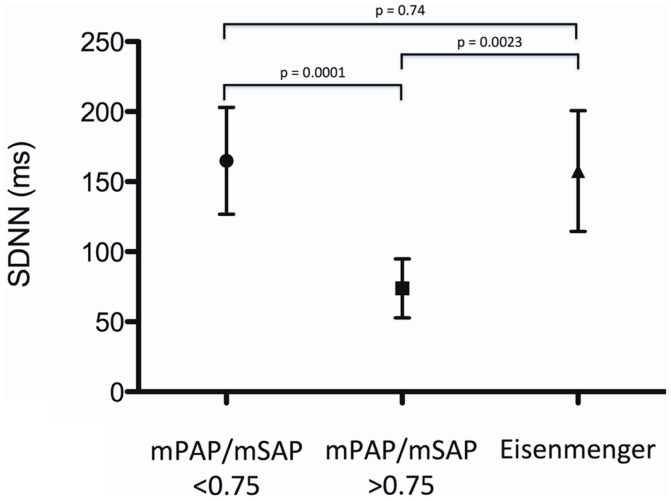
**Standard deviation of normal-to-normal intervals (SDNN) for patients with moderate PH (mPAP/mSAP ratio <0.75), patients with severe PH (mPAP/mSAP ratio >0.75) and patients with the Eisenmenger syndrome**.

Measures of HRV of patients with Eisenmenger syndrome were SDNN 157.6 ± 43.2 ms, SDANN 141.2 ± 45.3 ms, rMSSD 66.8 ± 16.0 ms, and pNN50 18.0 ± 11.6%, and were not significantly different compared to patients of group 1 (moderate PH) (*p* > 0.05 for all) (Figure 2).

All of the patients with Eisenmenger syndrome had ventricular premature beats [four of them (80%) more than 3/h]. About 40% of the patients had higher degreed ventricular arrhythmias, one had couplets of premature ventricular beats, and another patient had non-sustained ventricular tachycardia. One patient had frequent supraventricular premature beats and a non-sustained supraventricular tachycardia.

## Discussion

The results of our presented study showed for the first time that there is a strong relation between HRV and disease severity in children with PH. Except for patients with the Eisenmenger syndrome, HRV correlated with mPAP, mPAP/mSAP, PVRI, and PCWP. HRV may therefore serve as an additional marker of disease severity in children with PH without intracardiac shunt to identify patients at risk, to detect early clinical worsening, and to guide medical therapy.

There are different mechanisms discussed in the literature, which may contribute to impaired HRV in patients with PH. Stretching of the sinoatrial node may result in a reduction of HRV (9). Children with severe PH in our study had significantly larger right atriums than patients with moderate PH. Reduction of SDNN and SDANN can also be explained by chronic increased sympathetic nerve activity, which has been previously reported in adult patients with PAH (10). In addition, down-regulation of beta-adrenergic receptors has been shown to have impact on HRV in left and right ventricular failure (11).

Previous authors were able to demonstrate an impact of impaired left ventricular function on HRV (12) in patients with idiopathic dilated cardiomyopathy (IDC). In our study, left ventricular function was unimpaired in both subgroups. Furthermore, we were not able to demonstrate a relation between HRV and right ventricular function (assessed by TAPSE) and right atrial size (assessed by indexed right atrial area). Although TAPSE values were lower in the subgroup with severe PH, right ventricular function seemed to have no significant impact on HRV.

Remarkably, minimal heart rate was significantly higher in children with severe PH compared to children with moderate PH, and also mean heart rate tended to be higher, which might be a physiological response to achieve an adequate cardiac output despite of imminent RV failure. However, those patients with more accelerated heart rate may show a relevant shortening of diastole, and thus, impaired ventricular filling during diastole, which may result in progressive RV failure. β-Blockers may be a reasonable treatment option of this condition. β-Blockers can improve ventricular filling by decreasing heart rate and extending the duration of diastole. Concern about the use of β-blockers in PH was formerly based primarily on their potential to produce negative inotropic effects (13, 14). In animal models of PH, the non-selective β-blocker carvedilol seemed to improve right ventricular function and myocardial remodeling (15). However, further clinical studies in adults and children with PH are clearly needed.

The prevalence of serious supraventricular or ventricular arrhythmias in our PH-patients was low, and there was no relation of arrhythmias to disease severity. Previous studies on arrhythmias in patients with PH were focused on adult patients, and primarily revealed supraventricular arrhythmias (i.e., atrial flutter, atrial fibrillation) often leading to clinical deterioration (16–18). According to our results, supraventricular arrhythmias do not seem to be a relevant finding in the pediatric population.

Of particular interest are also the results of our adolescent patients with Eisenmenger syndrome. Eisenmenger patients are known for their more physiological adaptation to right ventricular afterload (19). As they have elevated pulmonary arterial pressures since the first days of life, their right ventricles usually show a remarkable hypertrophy, and right ventricular function is mostly preserved, in contrast to patients with severe IPAH and similar levels of pulmonary pressure, who develop increased right ventricular afterload later in life, which will frequently result in right ventricular failure (20, 21). Our study results showed a rather preserved HRV in patients with Eisenmenger syndrome despite of their systemic PA pressures, which might also reflect a more favorable autonomic adaptation. In contrast, remarkably high was the prevalence of relevant arrhythmias in the Eisenmenger group. This might be the reason for sudden cardiac death being not uncommon in patients with Eisenmenger syndrome (22).

### Study limitations

The small number of patients studied and the heterogeneity of the underlying etiology of PH may limit the interpretation of the data. Although there was a longer time interval between the HRV measurement and the invasive assessment of hemodynamics, the patients’ clinical condition remained stable and medical treatment unchanged, suggesting that the severity of PH did not change significantly during that time. While digoxin and beta blockers are known to alter heart rate and myocardial inotropy, which may affect the results of the HRV measurement, only three patients in this study received such medication.

## Conclusion

According to our results, children with severe PH may have alterations in HRV. Since HRV appears to be related to disease severity, it may therefore serve as an additional clinical marker of disease in PH. Remarkably, patients with the Eisenmenger syndrome seem to have preserved HRV despite of systemic PA pressures, which might reflect a more favorable autonomic adaptation.

## Conflict of Interest Statement

The authors declare that the research was conducted in the absence of any commercial or financial relationships that could be construed as a potential conflict of interest.
